# Editorial: How Children Learn From Parents and Parenting Others in Formal and Informal Settings: International and Cultural Perspectives

**DOI:** 10.3389/fpsyg.2020.01026

**Published:** 2020-06-26

**Authors:** Yvette R. Harris, Claudio Longobardi

**Affiliations:** ^1^Psychology, Miami University, Oxford, MS, United States; ^2^Department of Psychology, University of Turin, Turin, Italy

**Keywords:** parents, parenting, context, culture, learning, formal/informal setting

The goal of this special Research Topic is to bring together scholarship from diverse perspectives to address how broadly- and narrowly-defined parenting behaviors correlate with child and adolescent cognitive/emotional outcomes. Each contributor to this special Research Topic examines the issue of parenting behaviors and child/adolescent developmental outcomes from different methodological and theoretical orientations. The Research Topic includes 10 peer-reviewed articles, including 2 literature reviews and 8 empirical research articles. In accordance with the objectives of the topic, the contributions come from different nationalities, as parenting has a cultural component. However, while the literature agrees on the possible contribution of parenting to the adaptation of children and adolescents, influencing psychological well-being and cognitive and academic outcomes, little is known about cultural variables and about the association between constructs in different countries. Our contribution aims to stimulate debate in this direction, collecting contributions on the relationship between parenting and the adjustment of individuals at different points in development and, in particular, from different countries, in order to highlight the importance of the cultural context.

## Literature Review

In their article, Rollè et al. review the contemporary literature on father involvement and cognitive outcomes in preschool and middle school children. They conclude that father involvement is a multidisciplinary construct and that when, how, and why fathers are involved in their children's lives varies according to SES, ethnicity, education level, and residency. With a somewhat different focus, Trombetta et al. review the extant research on the linguistic environment of twins, with the goal of teasing apart the distinctive features of the home environment language, which potentially accounts for the language performance differences between twins and singletons. They point to the need to consider computational methods and contexts as we make interpretations about the differential linguistic performance between twins and singletons.

## Empirical Findings: Parenting and Cognitive Outcomes in Children and Adolescents

Suh et al. explore the interactions between mothers and their children from different ethnic backgrounds as they are engaged in block-building type tasks. We learn from their work, for example, that ethnicity is a significant predictor of maternal engagement and the time spent on tasks.

Lara and Saracostti provide a cultural perspective on levels of parental involvement and how these levels are associated with academic achievement in Chilean school age children, and they observed that highly- and medium-involved parents have children with higher academic outcomes.

Based upon the Walker et al. (2005) theoretical model, Jiang et al. extend our knowledge about the correlation between parental theories of intelligence (“incremental theory” vs. “entity theory”) and their involvement in children's education. They observed this association in China: a specific cultural context in which parents place an exceptionally high value on education and are actively engaged in their children's education at home. In particular, this study addressed the congruence and discrepancy between parents, highlighted the importance of parental beliefs in parent educational involvement, and revealed the significant role of mothers.

## Empirical Findings: Parenting and Emotional Outcomes/Mental Health in Children and Adolescents

In Australia, Waters et al. examine over time the relationship between strength-based parenting and subjective well-being in teens and preteens. Interestingly, the authors suggest that parenting is a significant predictor of the well-being of children in real time but that strength-based parenting does not predict children's future well-being. Considering the decline in strength-based parenting, the authors highlight the importance of supporting parent-child relationships during adolescence to improve their mental health.

Similarly, Calandri et al. found that adolescents with high levels of parental support are less depressed, and this finding holds central for adolescent girls, especially if the support is provided by their mothers. The researchers provide suggestions on designing intervention programs for adolescents and their parents.

The contribution of Bi et al. extends our knowledge about the association between parenting and parent-adolescent relationships in the Chinese cultural context. In particular, the novelty of this study is in providing some empirical evidence about the possible mediating role of beliefs regarding the legitimacy of parental authority and expectations of behavioral autonomy. The authors found that autonomy expectations mediated the effect of parenting style on parent-adolescent conflict, but authority legitimacy mediated the effect of parenting style on parent-adolescent cohesion. In addition, the results are discussed in light of gender differences.

In order to develop more sensitive prevention strategies for problematic mobile use of, Zhu et al. tested the possible mediating roles of perceived discrimination and school engagement in the relationship between parental rejection and problematic mobile use among Chinese university students. The results provide some empirical evidence about the possible role of parents in the development of problematic mobile use, and in particular, the results suggest that perceived discrimination and school engagement can exert sequential mediating effects on the path between parental rejection and problematic mobile phone use.

Finally, the interesting contribution of Hesp et al. demonstrates how seconds can be extended to real-time playful interaction between parents and children in the context of autism spectrum disorder. As commented by the authors, this “investigation opens the door toward the use of agent-based modeling as a cost-effective and ethical way to design and test new therapeutic interventions that stimulate the socio-emotional development of ASD children” (Hesp et al., p. 5).

In conclusion, the contributions presented in this topic tend to reconfirm, in different cultures, the role of parenting in promoting the adaptive development of children and adolescents, highlighting possible similarities but also differences between maternal and paternal function. Future studies will delve into this aspect, finding similarities and differences in the relationships between the contribution of parenting and, in particular, of mothers and fathers, to the psychological development and adaptation of children and adolescents from different cultural background contexts. In addition, the mechanisms that explain the link between parenting and the different constructs investigated can be studied in such contexts, taking into account possible variability on a cultural basis.

## Author Contributions

All authors listed have made a substantial, direct and intellectual contribution to the work, and approved it for publication.

## Conflict of Interest

The authors declare that the research was conducted in the absence of any commercial or financial relationships that could be construed as a potential conflict of interest.
